# Bayesian Perspective on Random Censored Survival Data

**DOI:** 10.1155/2014/430357

**Published:** 2014-10-29

**Authors:** Chris B. Guure, Samuel Bosomprah

**Affiliations:** Department of Biostatistics, School of Public Health, University of Ghana, Legon, Accra, Ghana

## Abstract

A unit is said to be randomly censored when the information on time occurrence of an event is not available due to either loss to followup, withdrawal, or nonoccurrence of the outcome event before the end of the study. It is assumed in independent random/noninformative censoring that each individual has his/her own failure time *T* and censoring time *C*; however, one can only observe the random vector, say, (*X*; *δ*). The classical approach is considered for analysing the generalised exponential distribution with random or noninformative censored samples which occur most often in biological or medical studies. The Bayes methods are also considered via a numerical approximation suggested by Lindley in 1980 and that of the Laplace approximation procedure developed by Tierney and Kadane in 1986 with assumed informative priors alongside linear exponential loss function and squared error loss function. A simulation study is carried out to compare the estimators proposed in this paper. Two datasets have also been illustrated.

## 1. Introduction

A new distribution for analysing time-to-event data was introduced by [1], known as generalised exponential distribution. Generalised exponential distribution can be used as an alternative to the well-known and used Weibull distribution in lifetime data analysis and reliability engineering according to [1].

The generalised exponential distribution has the distribution, density, and survival functions, respectively, as

(1) $$\begin{array}{c} F\left(t;\theta ,p\right)={\left\{1-\exp\left(-\theta t\right)\right\}}^{p},\;\theta ,p,t>0,\\ f\left(t;\theta ,p\right)=p\theta {\left\{1-\exp\left(-\theta t\right)\right\}}^{p-1}\exp\left(-\theta t\right),\\ S\left(t;\theta ,p\right)=1-{\left\{1-\exp\left(-\theta t\right)\right\}}^{p}, \end{array}$$

where *p* is the shape parameter and *θ* the scale parameter. Let the GE distribution with the shape parameter *p* and the scale parameter *θ* be denoted by GE(*θ*, *p*). According to [1], the two-parameter GE(*θ*, *p*) can be used quite effectively in analysing many lifetime data and can assume the place of the two-parameter gamma and two-parameter Weibull distributions. The two-parameter GE(*θ*, *p*) can have increasing and decreasing failure rates depending on the shape parameter.

Studies that involve time-to-event or survival data analysis are focussed on measuring time-to-event of an outcome. Time-to-event could vary from time to either death or the occurrence of a clinical endpoint such as disease or the attainment of a biochemical marker [2]. A special course of difficulty in the analysis of time-to-event data is the possibility that some individuals or units may not be observed for the full time to failure. In some circumstances, some individuals or units do not fail but are lost-to-followup during the observed period. Instead of knowing the failure time *t*, all we know about these individuals is that their time-to-failure exceeds some value, say *x*, where *x* is the follow-up time of these individuals in the study, which is referred to as censoring.

Under random or noninformative censoring, a sample of, say, *n*, elements are followed for some time, say *T*. An instance of this type of censoring occurs when the termination date for a medical trial is not fixed before the study starts but is rather chosen later, where the choice is influenced by the results of the study up to the termination time. In a straightforward overview of this scheme, which can be considered as time censoring, each element has a maximum inspection time of, say, *T*_*i*_, for *i* = 1,…, *n* which may possibly vary from one situation to another. Consider an experiment where we start with an observation of 50 cancer patients and terminate the experiment after a certain amount of time irrespective of the number of patients that have died or survived at the specified time. The survival of the patients may be due to withdrawal, inadequate monitoring mechanism, or deaths, which is not related to the purpose of the study.

Maximum likelihood estimator (MLE) is very popular both in the literature and in practice. Some researches have been done to compare MLE and the Bayesian approach in estimating the two parameters of the generalised distribution using hybrid and complete failure time data. Amongst them are [3], who studied generalized exponential distribution: Bayesian estimations. Reference [4] considered generalized exponential distribution by applying a different method of estimations. Other estimation procedures related to the above were considered by [5]. Reference [5] determined the Bayes estimates of the reliability function and the hazard rate of the Weibull failure time distribution by employing squared error loss function. Reference [6] studied Bayesian parameter and reliability estimate of Weibull failure time distribution; reference [7] studied the approximate Bayesian estimates for the Weibull reliability function and hazard rate from censored data by employing a new method that has the potential of reducing the number of terms in Lindley procedure. See also [8, 9]. Reference [10] studied Bayes estimators of modified Weibull distribution parameters using Lindley's approximation.

The main aim of this paper is to compare the classical maximum likelihood estimator to the proposed Bayesian estimators with two loss functions for the unknown parameters of the generalised exponential distribution for different sample sizes and parameter values.

## 2. Maximum Likelihood Estimation

Let (*t*_1_,…, *t*_*n*_) be the set of *n* random lifetimes with respect to the generalised exponential distribution with *p* and *θ* as the parameters, where *θ* is the scale parameter and *p* the shape parameter.

In random censoring as stated by [11], we assume *t*_*i*_ = min⁡(*T*_*i*_, *C*_*i*_), *δ* = 1 if *T*_*i*_ ≤ *C*_*i*_ and *δ* = 0 if *T*_*i*_ > *C*_*i*_. The observed data from *n* individuals is assumed to consist of the pair (*t*_*i*_, *δ*_*i*_), *i* = 1,…, *n*, so that the final result obtained will be the same provided *C*_*i*_ is available for all *i*.

It is therefore assumed that *T*⊥*C*; that is, *T* and *C* are independent of each other, which implies that the censoring time *C* is noninformative in analysing the failure time *T*. In order for this assumption to be valid, one has to ensure that the loss to follow-up of individuals is not as a result of the failure time defined. The likelihood function with respect to random censored data is

(2) $$\begin{array}{c} L\left(\delta ,t_{i};\theta ,p\right)=\overset{n}{\underset{i=1}{\prod }}f{\left(t_{i}\right)}^{\delta_{i}}S{\left(t_{i}\right)}^{1-\delta_{i}}, \end{array}$$

where *S*(·) is the survival function. Calculation of the maximum likelihood estimator often requires that some iterative (e.g., Newton-Raphson) procedures be implemented to obtain the parameters estimates. This can simply be obtained in any statistical software.

## 3. Bayesian Inference

In this section we consider the Bayes estimation of the two unknown parameters. Since both parameters are assumed to be greater than zero (0), we let both take on the following gamma prior distributions:

(3) $$\begin{array}{c} \pi_{1}\left(\theta \right)\propto \theta^{b-1}\exp\left(-\theta a\right),\;\theta >0,\\ \pi_{2}\left(p\right)\propto p^{d-1}\exp\left(-pc\right),\;p>0. \end{array}$$

Assume that the hyperparameters *a*, *b*, *c*, and *d* are known and >0. The joint density function of the data, *θ* and *p*, can be obtained as

(4) $$\begin{array}{c} \pi \left(\theta ,p,t,\delta \right)\propto l\left(\text{data}\;|\;\theta ,p\right)\pi_{1}\left(\theta \right)\pi_{2}\left(p\right). \end{array}$$

Bayesian inference is based on the posterior distribution which is given as

(5) $$\begin{array}{c} \pi^{*}\left(\theta ,p\;|\;t_{i},\delta_{i}\right)=\frac{\pi \left(\theta ,p,t,\delta \right)}{\iint_{0}^{\infty }\pi \left(\theta ,p,t,\delta \right)d\theta \;dp}. \end{array}$$

The ratio of the two integrals given in (5) cannot be obtained in a closed form. We can apply a numerical integration technique, which may be computationally intensive especially in high-dimensional parameter space. It is also possible to make use of numerical approximation methods such as [12] and/or [9]. In this paper, we shall consider both methods for this type of censoring scheme and for this distribution, since we are unaware of any study employing both methods for this distribution and with this type of censored data apart from the former by [3] with uncensored data. This approach is considered under two loss functions, namely, LINEX and squared error loss.

### 3.1. Lindley Approximation

Reference [12] proposed a procedure to approximate the ratio of integrals. This approach has been used by several authors like [3, 6] to obtain the approximate Bayes estimators. Reference [12] shows the approximate procedure for evaluating ratio of integrals of the form

(6) $$\begin{array}{c} E\left\{u\left(\alpha \right)\;|\;x\right\}=\frac{\int \omega \left(\alpha \right)\exp\left[l\left(\alpha \right)\right]d\alpha }{\int v\left(\alpha \right)\exp\left[l\left(\alpha \right)\right]d\alpha }, \end{array}$$

where *α* = (*α*_1_, *α*_2_,…, *α*_*m*_) and *ℓ*(*α*) is the logarithm of the likelihood function and *ω*(*α*), *v*(*α*) are arbitrary functions of *α*. Assume that *v*(*α*) is the prior distribution for *α* and *ω*(*α*) = *u*(*α*) · *v*(*α*) with *u*(*α*) being some function of interest. The posterior expectation of *q*(*α*) is given as

(7) $$\begin{array}{c} E\left\{q\left(\alpha \right)\;|\;t\right\}=\frac{\int u\left(\alpha \right)\exp\left\{l\left(\alpha \right)+\rho \left(\alpha \right)\right\}d\alpha }{\int \exp\left\{l\left(\alpha \right)+\rho \left(\alpha \right)\right\}d\alpha }, \end{array}$$

where *ρ*(*α*) = log⁡*v*(*α*). An outline of the procedure can be obtained from [12] and a recent paper by [13]. Lindley's procedure can be approximated asymptotically by

(8) Euα ∣ x=u+12∑i∑juij+2ui·ρj·σij  +12∑i∑j∑k∑llijk·σij·σkl·ul.

Considering the Bayesian estimator under the squared error loss function, which is the posterior mean, the following can be obtained where *u*_1_ and  *u*_11_ are the first and second derivatives of the scale parameter (*θ*) while *u*_2_ and *u*_22_ are also the first and second derivatives of (*p*):

(9) uθ=θ,  u1=1,  u11=0,up=p,  u2=1,  u22=0,ρ=ln⁡π1θ+ln⁡π2p,ρ1=b−1θ−a,  ρ2=d−1p−c.σ11=−l20−1,  σ22=−l02−1.

Refer to appendix section for derivatives with respect to the shape and scale parameters.

### 3.2. Linear Exponential Loss Function with Lindley Procedure

Unlike the symmetric loss function (squared error), this loss function measures the degree of underestimation and overestimation of the estimated parameter.

The Bayes estimator of, say, *α*, which is denoted by ${\hat{\alpha }}_{\text{BL}}$ under LINEX loss function, is

(10) $$\begin{array}{c} {\hat{\alpha }}_{\text{BL}}=-\frac{1}{k}\ln\left\{E_{\alpha }\left[\exp\left(-k\alpha \right)\right]\right\}, \end{array}$$

provided that *E*_*α*_[exp⁡(−*kα*)] exists and is finite.

The Bayes estimator ${\hat{u}}_{\text{BL}}$ of a function *u*_BL_ = *u*[exp⁡(−*kθ*), exp⁡(−*kp*)] under LINEX is given as

(11) $$\begin{array}{c} {\hat{u}}_{\text{BL}}=\frac{\iint u_{\text{BL}}\pi_{1}\left(\theta \right)\pi_{2}\left(p\right)L\left(\delta ,t_{i};\theta ,p\right)d\theta \;dp}{\iint \pi_{1}\left(\theta \right)\pi_{2}\left(p\right)L\left(\delta ,t_{i};\theta ,p\right)d\theta \;dp}, \end{array}$$

where

(12) $$\begin{array}{c} \hat{\theta }=\exp\left(-k\theta \right),\;\;u_{1}=\frac{\partial u}{\partial \theta }=-k\exp\left(-k\theta \right),\\ u_{11}=\frac{\partial^{2}u}{\partial \theta^{2}}=k^{2}\exp\left(-k\theta \right),\;\;u_{2}=u_{22}=0,\\ \hat{p}=\exp\left(-kp\right),\;\;u_{2}=\frac{\partial u}{\partial p}=-k\exp\left(-kp\right),\\ u_{22}=\frac{\partial^{2}u}{\partial p^{2}}=k^{2}\exp\left(-kp\right),\;\;u_{1}=u_{11}=0. \end{array}$$

### 3.3. Tierney and Kadane

Observing from Section 3.1, it is clear that the Lindley approach demands or requires that we evaluate the third derivatives of the likelihood function. Depending on the distribution and the number of parameters involved, this approach can be very difficult to achieve. Tierney and Kadane through Laplace approximation procedure gave an alternative to the Lindley approach which only requires the first and second derivatives of the likelihood function. Let *L*(*α*; *t*) be the likelihood function of *α* based on *n* number of observations. *π*(*α*) represents the prior distribution defined over the parameter space, *v*(*α*) represents the loss function, and *q*(*α*∣*t*) represents the posterior distribution of *α*. The Bayes estimate of a function *q*(*α*) under the squared error loss function is the posterior mean and is given as

(13) $$\begin{array}{c} \hat{q}=E\left\{q\left(\alpha \right)\;|\;t\right\}=\frac{\int \exp\left(nL^{*}\left(\alpha \right)d\alpha \right)}{\int \exp\left(nL\left(\alpha \right)d\alpha \right)}, \end{array}$$

with

(14) $$\begin{array}{c} L\left(\alpha \right)=\frac{\log\pi \left(\alpha \right)+\log L\left(t\;|\;\alpha \right)}{n},\\ L^{*}\left(\alpha \right)=\frac{\log v\left(\alpha \right)+\log\pi \left(\alpha \right)+\log L\left(t\;|\;\alpha \right)}{n}. \end{array}$$

Equation (13) can be approximated in the form

(15) $$\begin{array}{c} {\hat{q}}_{\text{BS}}={\left[\frac{\left|\sum_{}^{*}\right|}{\left|\sum \right|}\right]}^{1/2}\exp\left\{n\left[L^{*}\left({\hat{\alpha }}^{*}\right)-L\left(\hat{\alpha }\right)\right]\right\}. \end{array}$$

This can similarly be expressed as

(16) $$\begin{array}{c} {\hat{q}}_{\text{BS}}={\left[\frac{\left|\sum_{}^{*}\right|}{\left|\sum \right|}\right]}^{1/2}\frac{q\left({\hat{\alpha }}^{*}\right)q\left({\hat{\alpha }}^{*}\;|\;t\right)}{q\left(\hat{\alpha }\;|\;t\right)}, \end{array}$$

where ${\hat{\alpha }}^{*}$ and $\hat{\alpha }$ maximize *ℓ* ^*^(*α* ^*^) and *ℓ*(*α*), respectively, and ∑^*^ and ∑ are the negatives of the inverse Hessians of *ℓ* ^*^ and *ℓ*, respectively.

The matrix ∑ takes the form

(17) $$\begin{array}{c} \sum =\left[\left(-\frac{\partial^{2}l}{\partial^{2}\theta }\times -\frac{\partial^{2}l}{\partial^{2}p}\right)-\left(-\frac{\partial^{2}l}{\partial \theta \partial p}\times -\frac{\partial^{2}l}{\partial p\partial \theta }\right)\right]. \end{array}$$

We can similarly obtain the expression for the matrix ∑^*^, which involves the partial derivatives of *ℓ* ^*^. In applying the method the following need to be maximised:

(18) l=1nLδ,ti;θ,pa−1ln⁡θ−θb+c−1ln⁡p   −pd+log⁡Lδ,ti;θ,p

(19) l∗=1nLδ,ti;θ,pln⁡θ+ln⁡p+a−1ln⁡θ−θb+c−1ln⁡p   −pd+log⁡Lδ,ti;θ,p.

Setting ∂*ℓ*/∂*θ* and ∂*ℓ*/∂*p* to zero produces the following system of equations:

(20) $$\begin{array}{c} \frac{\partial l}{\partial \theta }=\frac{1}{n}\left\{\frac{\left(a-1\right)}{\theta }-b+\frac{\partial \text{Log}L\left(\theta ,p;t_{i},\delta_{i}\right)}{\partial \theta }\right\},\\ \frac{\partial l}{\partial p}=\frac{1}{n}\left\{\frac{\left(c-1\right)}{p}-d+\frac{\partial \text{Log}L\left(\theta ,p;t_{i},\delta_{i}\right)}{\partial p}\right\}, \end{array}$$

where ∂Log*L*(*θ*, *p*; *t*_*i*_, *δ*_*i*_)/∂*θ* and ∂Log*L*(*θ*, *p*; *t*_*i*_, *δ*_*i*_)/∂*p* are easy to obtain. Refer to Appendix section for ∂^2^ *ℓ*/∂*θ* ^2^, ∂^2^ *ℓ*/∂*p* ^2^, and ∂^2^ *ℓ*/∂*θ*∂*p*.

### 3.4. Linear Exponential Loss Function with Tierney and Kadane Procedure

The Bayesian estimator ${\hat{u}}_{\text{BL}}$ of a function *u*_BL_ = *u*[exp⁡(−*kθ*), exp⁡(−*kp*)] under LINEX with respect to Tierney and Kadane procedure is given as

(21) $$\begin{array}{c} {\hat{u}}_{\text{BL}}=\frac{\iint u_{\text{BL}}\pi_{1}\left(\theta \right)\pi_{2}\left(p\right)L\left(\delta ,t_{i};\theta ,p\right)d\theta \;dp}{\iint \pi_{1}\left(\theta \right)\pi_{2}\left(p\right)L\left(\delta ,t_{i};\theta ,p\right)d\theta \;dp}, \end{array}$$

where

(22) l=1na−1ln⁡θ−θb+c−1ln⁡p−pd  +log⁡Lδ,ti;θ,p,l∗=1nln⁡exp⁡−kθ+ln⁡exp⁡−kp+a−1ln⁡θ   −θb+c−1ln⁡p−pd+log⁡Lδ,ti;θ,p.

The same approach is also adopted with the squared error loss function to obtain the Bayes estimates of the unknown parameters.

## 4. Data Source

### 4.1. Data 1

The data for this example are on survival of patients with cervical cancer, recruited to a randomised trial aimed at analysing the effect of addition of a radio sensitiser to radiotherapy (new therapy—“treatment B”) compared to using only radiotherapy (control—“treatment A”). Treatment A was given to 16 and treatment B to 14 patients. The data are in days since the start of the study; the event of interest is death caused by this cancer. Our main interest is on the patients under treatment A, which is fairly small to illustrate the proposed methods in this paper. The data is obtained from [14]. Starred observations are censored: 90, 890^*^, 142, 1037, 150, 1090^*^, 269, 1113^*^, 291, 1153, 468^*^, 1297, 680, 1429, 837, 1577^*^. The results are depicted in Table 3.

### 4.2. Data 2

The following data which are considered large are obtained from [11]. The data represent survival times for 121 breast cancer patients who were treated over the period 1929–1938. Times are in months and asterisks denote censoring times: 0.3, 0.3^*^, 4.0^*^, 5.0, 5.6, 6.2, 6.3, 6.6, 6.8, 7.4^*^, 7.5, 8.4, 8.4, 10.3, 11.0, 11.8, 12.2, 12.3, 13.5, 14.4, 14.4, 14.8, 15.5^*^, 15.7, 16.2, 16.3, 16.5, 16.8, 17.2, 17.3, 17.5, 17.9, 19.8, 20.4, 20.9, 21.0, 21.0, 21.1, 23.0, 23.4^*^, 23.6, 24.0, 24.0, 27.9, 28.2, 29.1, 30, 31, 31, 32, 35, 35, 37^*^, 37^*^, 37^*^, 38, 38^*^, 38^*^, 39^*^, 39^*^, 40, 40^*^, 40^*^, 41, 41, 41^*^, 42, 43^*^, 43^*^, 43^*^, 44, 45^*^, 45^*^, 46^*^, 46^*^, 47^*^, 48, 49^*^, 51, 51, 51^*^, 52, 54, 55^*^, 56, 57^*^, 58^*^, 59^*^, 60, 60^*^, 60^*^, 61^*^, 62^*^, 65^*^, 65^*^, 67^*^, 67^*^, 68^*^, 69^*^, 78, 80, 83^*^, 88^*^, 89, 90, 93^*^, 96^*^, 103^*^, 105^*^, 109^*^, 109^*^, 111^*^, 115^*^, 117^*^, 125^*^, 126, 127^*^, 129^*^, 129^*^, 139^*^, 154^*^.

## 5. Simulation Study

Since it is difficult to compare the performance of the proposed methods theoretically, we have performed an extensive simulation to compare the estimators through mean squared errors and absolute biases by employing different sample sizes with different parameter values. We considered a sample size of *n* = 25, 50, and 100. The following steps were employed to generate the data. The generation of GE(*θ*, *p*) is simple as stated in [4]. If *U* follows a uniform distribution in the interval [0,1], then *Y* = (−ln⁡(1 − *U* ^(1/*p*)^)/*θ*) follows GE(*θ*, *p*). Consequently, with a very good uniform random number generator, the generation of GE(*θ*, *p*) random deviate is immediate.

A lifetime *T* is generated from the sample sizes indicated above from the GE(*θ*, *p*) distribution which represent failure of the product. The values of the assumed actual shape parameter (*p*) of the GE(*θ*, *p*) distribution were taken to be 0.8, 1.2 and 2.0. The scale parameter (*θ*) was considered throughout to be 1 without loss of generality. The same sample size is generated from the uniform distribution for the censored time *C* with (0, *b*), where the value of *b* depends solely on the proportion of the observations that are censored. In our study, we considered the percentage of censoring to be 25. *t*_*i*_ = min⁡(*T*_*i*_, *C*_*i*_) is taken as the minimum of the failure time and that of the censored time of the observed time *T*. To compute the Bayes estimates, an assumption is made such that *θ* and *p* take, respectively, Gamma(*a*, *b*) and Gamma(*c*, *d*) priors. We set the hyperparameters to 0; that is, *a* = *b* = *c* = *d* = 0; this makes the priors noninformative. The values of the loss parameter for the LINEX loss function are *k* = ±0.7. These were iterated 1000 times. The mean squared errors and the absolute biases are determined and presented for the purpose of comparison.

## 6. Results and Discussion

The main objective of this study is to obtain the estimates of the generalised exponential distribution parameters and compare the proposed methods applied in this paper. In order to examine the estimates of the parameters which cannot be obtained analytically, we made use of different numerical approximation procedures and have obtained absolute biases and mean squared errors of the estimated parameters.

**Figure 1:**
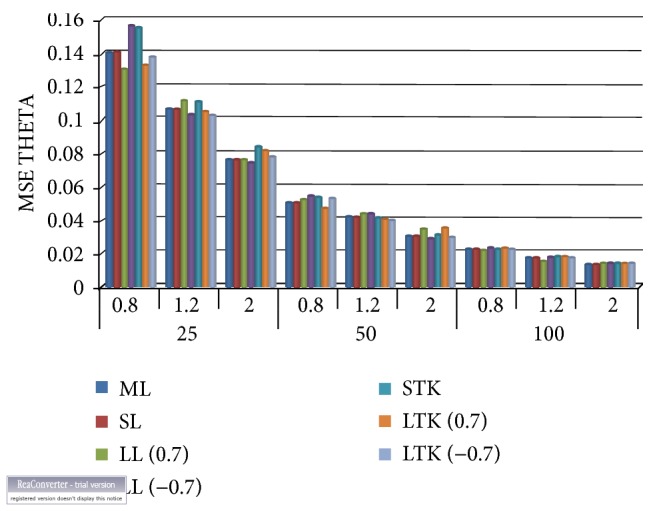
Mean squared errors for the scale parameter. ML: maximum likelihood, SL: squared error loss under Lindley approximation, LL: LINEX loss under Lindley, STK: squared error under Tierney and Kadane method, and LTK: LINEX loss under Tierney and Kadane method.

**Figure 2:**
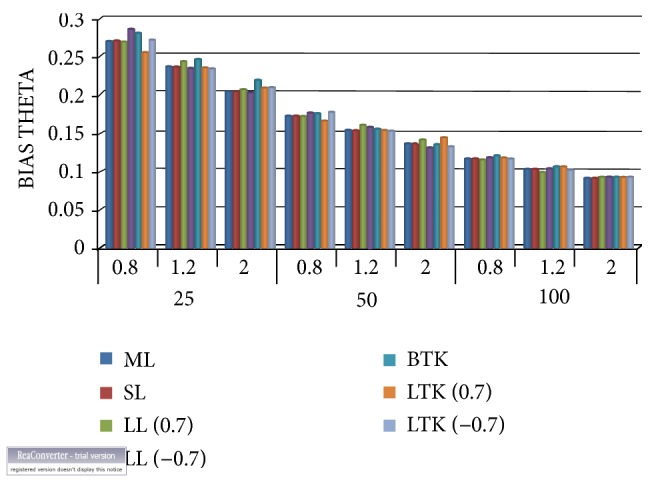
Absolute bias for the scale parameter. ML: maximum likelihood, SL: squared error loss under Lindley approximation, LL: LINEX loss under Lindley, STK: squared error under Tierney and Kadane method, and LTK: LINEX loss under Tierney and Kadane method.

Observing from Table 1 and Figures 1 and 2, it is evident that the smallest mean squared errors vis-$\overset{-}{a}$-vis the absolute biases for the estimated scale parameter $\left(\hat{\theta }\right)$ occurred under the Bayesian estimator with the linear exponential loss function. The loss parameter from which we obtained the smallest mean squared errors is 0.7, which is above zero, implying this approach is preferred if overestimation is more serious than underestimation. This occurred largely with the Lindley numerical approximation procedure, followed by Tierney and Kadane. As the sample size increases, all the estimators' mean squared errors correspondingly decrease. Another observation made that needs to be mentioned is that Lindley approximation method under the squared error loss function performed better than that of the Tierney and Kadane with respect to the generalised exponential scale parameter. As illustrated clearly in Figure 2, both Tierney and Kadane had equal minimum absolute biases.

**Figure 3:**
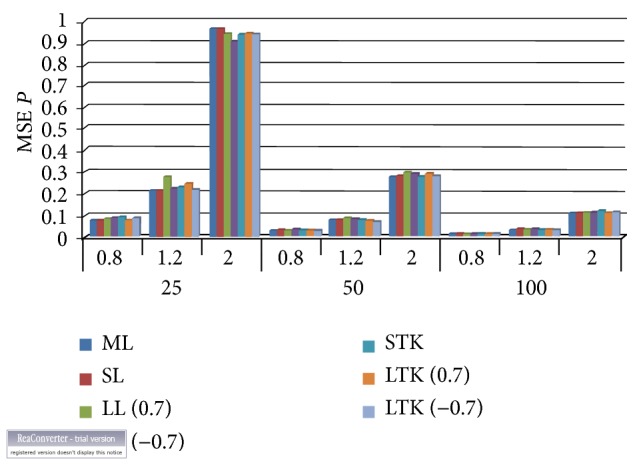
Mean squared errors for the shape parameter. ML: maximum likelihood, SL: squared error loss under Lindley approximation, LL: LINEX loss under Lindley, STK: squared error under Tierney and Kadane method, and LTK: LINEX loss under Tierney and Kadane method.

**Figure 4:**
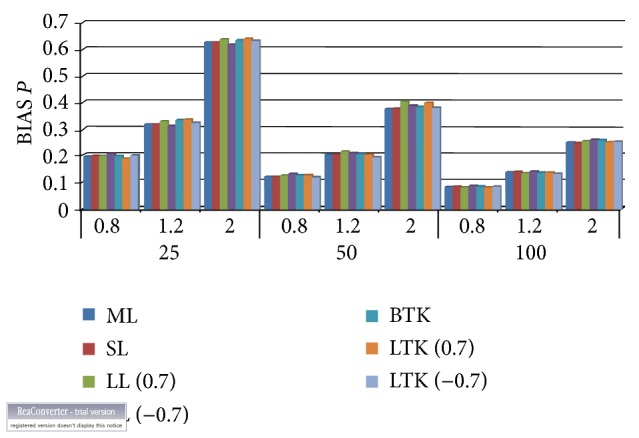
Absolute bias for the shape parameter. ML: maximum likelihood, SL: squared error loss under Lindley approximation, LL: LINEX loss under Lindley, STK: squared error under Tierney and Kadane method, and LTK: LINEX loss under Tierney and Kadane method.

Considering Table 2 alongside Figures 3 and 4, which contain the mean squared errors and the absolute biases of the estimated shape parameter $\left(\hat{p}\right)$, we noticed that the Bayesian estimator under the Tierney and Kadane method performed better than the Lindley approach but maximum likelihood estimator overall had the smallest mean squared error followed by Tierney and Kadane. The minimum absolute bias occurred predominantly with the Tierney and Kadane approach. The bold numbers indicate the smallest and minimum biases of the estimated parameters with their corresponding estimators. The Bayes estimator with Lindley numerical approximation procedure for the exponential distribution performed better under the squared error loss function for the shape parameter than that of the Tierney and Kadane numerical method to a very large extent. All the estimators' mean squared errors got closer as the sample size increased.

The Bayesian estimator under linear exponential loss function with the positive loss parameter has the smallest standard error as illustrated in Table 3. This happened with the approximation procedure suggested by Tierney and Kadane; it implies that linear exponential loss function overestimates the scale and shape parameters of the generalised exponential distribution. From this example, where the sample size is considered to be fairly small, we noticed that using the Tierney and Kadane approach via Bayes under squared error loss performs fairly better than that of the Lindley method as well as the maximum likelihood estimator.

Using the iterative procedure suggested in this paper for both MLEs and Bayes with respect to data 2, the MLEs of $\hat{\theta }$ and $\hat{p}$ are 0.765027 and 6.277847 with their corresponding standard errors as 0.006323 and 0.010377. Since we do not have any prior information on the hyperparameters, we assume *a* = *b* = *c* = *d* = 0. This makes the priors on $\hat{\alpha }$ and $\hat{\beta }$ noninformative. For computing the Bayes estimators, we considered the squared error loss and linear exponential loss functions and gamma priors on both *α* and *β* same as the approach used in the simulation section. After computing the Bayes estimators via Lindley approximation procedure under squared error loss for $\hat{\theta }$ and $\hat{p}$, the following parameters estimates and standard errors were obtained, respectively, 0.765027, 6.277847 and 0.006325, 0.010376.

Computing the Bayes estimates of $\hat{\theta }$ and $\hat{p}$ and their corresponding standard errors under the linear exponential loss function with a loss parameter of 0.7, we have 0.765029, 6.277860 and 0.006323, 0.010377. With the loss parameter being −0.7, we have 0.765025, 6.277840 and 0.006323, 0.010376, respectively. Considering 95% confidence interval of MLE, we have $\hat{\theta }$ = (0.752634, 0.777419) and $\hat{p}$ = (6.257508, 6.298185). Bayes credible intervals under squared error loss function of $\hat{\theta }$ and $\hat{p}$ are 0.752634, 0.777419 and 6.257508, 6.298185, respectively. The Bayes credible intervals with respect to the LINEX loss function with the loss parameter 0.7 for $\hat{\theta }$ and $\hat{p}$ are 0.752637, 0.777421 and 6.257521, 6.298198 and those of the −0.7 are 0.752633, 0.777417 and 6.257501, 6.298178, respectively.

Computing the Bayes estimators using Tierney and Kadane (T & K) approximation procedure under squared error loss function for $\hat{\theta }$ and $\hat{p}$, we have, respectively, the following parameters estimates and standard errors: 0.764725, 6.275374 and 0.006320, 0.010373. Calculating the Bayes estimates via Tierney and Kadane of $\hat{\theta }$ and $\hat{p}$ with their corresponding standard errors under the linear exponential loss function with a loss parameter of +0.7, we have 0.765633, 6.282807 and 0.006328, 0.010385. With the loss parameter of −0.7, we have 0.763671, 6.277809 and 0.006311, 0.010358, respectively. Bayes credible intervals using Tierney and Kadane under squared error loss function of $\hat{\theta }$ and $\hat{p}$ are 0.752338, 0.777113 and 6.255044, 6.295704. The Bayes credible intervals with respect to the LINEX loss function with the loss parameter +0.7 for $\hat{\theta }$ and $\hat{p}$ are 0.753231, 0.778035 and 6.262453, 6.303161 and those of −0.7 are 0.751301, 0.776041 and 6.246441, 6.287045, respectively.

As clearly stipulated above, the estimator with the smallest standard error is Bayesian under the linear exponential loss function for both the scale and shape parameters. This happened under the Tierney and Kadane numerical approximation procedure. This is followed by Bayes estimator using the squared error loss function, again with the Tierney and Kadane method. We observed that the linear exponential loss function had the narrowest credible intervals with respect to the Tierney and Kadane approach as compared to the credible intervals of Bayes using Lindley and the confidence intervals obtained from maximum likelihood estimator. This happened with a negative loss parameter, an indication of underestimation of the generalised exponential distribution parameters.

## 7. Conclusion

From the results and discussions above it is evident that the Bayesian estimator under linear exponential loss function performed quite better well than Bayes under squared error loss function and maximum likelihood estimator for estimating both the scale parameter $\left(\hat{\theta }\right)$ and shape parameter $\hat{p}$, with both MSE and absolute bias. Lindley method performed better than T & K for the scale parameter with regard to mean squared errors while T & K performed better for the shape parameter with both the mean squared errors and the absolute bias. Considering the standard errors obtained for the real data analysis, we can state that the T & K method outperformed the Lindley numerical approximation and the maximum likelihood estimator.

## Figures and Tables

**Table 1:** Average mean squared errors and absolute biases for $\left(\hat{\theta }\right)$.

*n*			*β* = 0.8	*β* = 1.2	*β* = 2.0
25		ML	0.140450 (0.270431)	0.106852 (0.237027)	0.076700 (0.204796)

	Lindley	BS	0.140834 (0.271052)	0.106857 (0.237097)	0.076705 (0.204866)
		BL (*k* = 0.7)	**0.131177** (0.269803)	0.111975 (0.244191)	0.076627 (0.207483)
		BL (*k* = −0.7)	0.156620 (0.285894)	0.103548 (0.235582)	**0.074685 (0.203569)**

	T & K	BS	0.155934 (0.281566)	0.111042 (0.247333)	0.084400 (0.219475)
		BL (*k* = 0.7)	0.133596 ** (0.256556) **	0.105219 (0.235598)	0.082096 (0.210066)
		BL (*k* = −0.7)	0.137543 (0.272175)	**0.103369 (0.234812)**	0.078827 (0.210891)

50		ML	0.050784 (0.173185)	0.042238 (0.153893)	0.030681 (0.137025)

	Lindley	BS	0.050786 (0.173238)	0.042239 (0.153932)	0.030895 (0.137487)
		BL (*k* = 0.7)	0.052969 (0.171820)	0.044209 (0.160487)	0.033233 (0.141801)
		BL (*k* = −0.7)	0.054932 (0.177022)	0.043975 (0.158316)	**0.028858 (0.131245)**

	T & K	BS	0.054196 (0.176032)	0.041590 (0.156810)	0.031042 (0.134974)
		BL (*k* = 0.7)	**0.047443 (0.166083)**	0.040373 (0.153958)	0.035063 (0.144515)
		BL (*k* = −0.7)	0.053519 (0.178140)	**0.040125 (0.153786)**	0.029654 (0.132043)

100		ML	0.023217 (0.117084)	0.017736 (0.103513)	**0.013552 (0.091643)**

	Lindley	BS	0.023217 (0.117092)	0.017736 (0.103531)	0.013572 (0.091785)
		BL (*k* = 0.7)	**0.022439 (0.115258)**	**0.016563 (0.099769)**	0.014159 (0.093593)
		BL (*k* = −0.7)	0.023443 (0.119925)	0.018040 (0.104651)	0.014358 (0.094881)

	T & K	BS	0.023403 (0.121568)	0.018424 (0.106916)	0.014604 (0.093797)
		BL (*k* = 0.7)	0.023223 (0.118237)	0.018637 (0.107096)	0.014348 (0.094252)
		BL (*k* = −0.7)	0.023205 (0.118180)	0.017766 (0.102358)	0.014784 (0.095141)

ML: maximum likelihood, BL: LINEX loss function, BS: squared error loss function.

**Table 2:** Average mean squared errors and absolute bias for $\left(\hat{p}\right)$.

*n*			*β* = 0.8	*β* = 1.2	*β* = 2.0
25		ML	**0.074937** (0.196749)	**0.210881** (0.317231)	0.969396 (0.631983)

	Lindley	BS	0.074946 (0.196841)	0.210886 (0.317302)	0.969470 (0.632254)
		BL (*k* = 0.7)	0.083694 (0.199075)	0.274929 (0.329363)	0.942216 (0.639469)
		BL (*k* = −0.7)	0.088910 (0.207179)	0.222206 ** (0.311704) **	**0.908126 (0.618789)**

	T & K	BS	0.090567 (0.198705)	0.230426 (0.333524)	0.936865 (0.634128)
		BL (*k* = 0.7)	0.075149 ** (0.188331) **	0.245118 (0.335657)	0.944325 (0.639520)
		BL (*k* = −0.7)	0.087542 (0.201015)	0.217948 (0.323301)	0.938991 (0.635110)

50		ML	0.026631 (0.121009)	0.076945 (0.204643)	**0.276000 (0.375176)**

	Lindley	BS	0.026631 (0.121018)	0.076950 (0.204715)	0.280979 (0.377409)
		BL (*k* = 0.7)	0.028234 (0.125414)	0.086934 (0.217802)	0.297106 (0.403637)
		BL (*k* = −0.7)	0.034478 (0.132509)	0.079751 (0.210635)	0.289681 (0.387573)

	T & K	BS	0.028420 (0.127050)	0.077981 (0.207406)	0.276088 (0.382376)
		BL (*k* = 0.7)	0.028553 (0.125712)	0.073419 (0.206062)	0.290441 (0.399910)
		BL (*k* = −0.7)	**0.026399 (0.120282)**	**0.068112 (0.195011)**	0.278232 (0.379734)

100		ML	0.011790 (0.083599)	0.031119 (0.137169)	**0.106223 (0.250598)**

	Lindley	BS	0.011825 (0.083787)	0.031133 (0.137289)	0.106233 (0.250603)
		BL (*c* = 0.7)	**0.010882** (0.081341)	0.029755 (0.134297)	0.109028 (0.255089)
		BL (*c* = −0.7)	0.012339 (0.085559)	0.032030 (0.138596)	0.111296 (0.259628)

	T & K	BS	0.011880 (0.084372)	0.030722 (0.135511)	0.115963 (0.258207)
		BL (*k* = 0.7)	0.011434 ** (0.081271) **	0.031457 (0.134959)	0.108760 (0.251214)
		BL (*k* = −0.7)	0.011318 (0.083157)	**0.029563 (0.132158)**	0.112661 (0.253481)

ML: maximum likelihood, BL: LINEX loss function, BS: squared error loss function.

**Table 3:** Standard errors for $\hat{\alpha }$ and $\hat{\beta }$ with *n* = 16.

	MLE	Lindley			T & K		
		BS	BL	BL	BS	BL	BL
			*k* = 0.7	*k* = −0.7		*k* = 0.7	*k* = −0.7
$\hat{\theta }$	0.011977	0.011970	0.011977	0.011979	0.011968	**0.011952**	0.011954
s.e $\left(\hat{\theta }\right)$	0.000749	0.000748	0.000749	0.000750	0.000748	**0.000747**	0.000747
$\hat{p}$	0.244102	0.244831	0.244763	0.244954	0.243901	**0.243897**	0.245650
s.e $\left(\hat{p}\right)$	0.003051	0.003060	0.003059	0.003062	0.003049	**0.003048**	0.003071

## Appendix

Let the following assumptions hold under the Lindley approach:

(A.1) e=∑i=1n1−1−exp⁡−θtip,f=∑i=1n1−exp⁡−θti,g=exp⁡−θ∑i=1nti,l20=−∑i=1nδiθ2−p−1∑i=1nδi∑i=1nti2gf −p−1∑i=1nδi∑i=1nti2g2f2 −1−∑i=1nδifpp2∑i=1nti2g2f2e +1−∑i=1nδifpp∑i=1nti2gfe +1−∑i=1nδifpp∑i=1nti2g2f2e −1−∑i=1nδifp2p2∑i=1nti2g2f2e2,l30=2∑i=1nδiθ3+p−1∑i=1nδi∑i=1nti3gf +3p−1∑i=1nδi∑i=1nti3g2f2 +2p−1∑i=1nδi∑i=1nti3g3f3 −1−∑i=1nδifpp3∑i=1nti3g3f3e +31−∑i=1nδifpp2∑i=1nti3g2f2e +31−∑i=1nδifpp2∑i=1nti3g3f3e −31−∑i=1nδifp2p3∑i=1nti3g3f3e2 −1−∑i=1nδifpp∑i=1nti3gfe −31−∑i=1nδifpp∑i=1nti3g2f2e +31−∑i=1nδifp2p2∑i=1nti3g2f2e2 −21−∑i=1nδifpp∑i=1nti3g3f3e +31−∑i=1nδifp2p2∑i=1nti3g3f3e2 −21−∑i=1nδifp3p3∑i=1nti3g3f3e3,l02=−∑i=1nδip2−1−∑i=1nδifplog⁡f2e −1−∑i=1nδifp2log⁡f2e2,l03=2∑i=1nδip3−1−∑i=1nfplog⁡f3e −31−∑i=1nδifp2log⁡f3e2 −21−∑i=1nδifp3log⁡f3e3.

Note that *ℓ*_20_ and *ℓ*_30_ are the second and third derivatives for the scale parameter of the log-likelihood function while *ℓ*_02_ and *ℓ*_03_ are the derivatives of the shape parameter.

Using 20 from the Tierney and Kadane numerical approach, the following derivatives are obtained:

(A.2) ∂2l∂θ2=1n1−∑i=1nδifp2p2∑i=1nti2g2f2e2−a−1−∑i=1nδiθ2   −p−1∑i=1nδi∑i=1nti2gf   −p−1∑i=1nδi∑i=1nti2g2f2   −1−∑i=1nδifpp2∑i=1nti2g2f2e   +1−∑i=1nδifpp∑i=1nti2gfe   +1−∑i=1nδifpp∑i=1nti2g2f2e   −1−∑i=1nδifp2p2∑i=1nti2g2f2e2,∂2l∂p2=1n1−∑i=1nδifp2log⁡f2e2−c−1−∑i=1nδip2   −1−∑i=1nδifplog⁡f2e   −1−∑i=1nδifp2log⁡f2e2,∂2l∂θ∂p =1n×fe2−1exp⁡−θ∑i=1ntiln⁡(f)∑i=1nδi∑i=1ntiexp⁡−θ∑i=1ntif    −exp⁡−θ∑i=1ntiln⁡fexp⁡−θ∑i=1nti1−∑i=1nδifpp∑i=1nti      ×exp⁡−θ∑i=1ntiln⁡f×fe−1    −exp⁡−θ∑i=1ntiexp⁡−θ∑i=1nti1−∑i=1nδifp∑i=1nti      ×exp⁡−θ∑i=1nti×fe−1    −exp⁡−θ∑i=1ntiln⁡(f)exp⁡−θ∑i=1nti1−∑i=1nδifp2p∑i=1nti       ×exp⁡−θ∑i=1ntiln⁡(f)      ×fe2−1exp⁡−θ∑i=1ntiln⁡⁡f∑i=1nδi∑i=1ntiexp⁡−θ∑i=1ntif.

By employing 19, the following derivatives are obtained:

(A.3) $$\begin{array}{c} \frac{\partial^{2}l^{*}}{\partial \theta^{2}}=\frac{1}{n}\left[-\frac{1}{\theta^{2}}\right]+\frac{\partial^{2}l}{\partial \theta^{2}},\\ \frac{\partial^{2}l^{*}}{\partial p^{2}}=\frac{1}{n}\left[-\frac{1}{p^{2}}\right]+\frac{\partial^{2}l}{\partial p^{2}}\\ \frac{\partial^{2}l^{*}}{\partial \theta \partial p}=\frac{\partial^{2}l^{*}}{\partial p\partial \theta }=\frac{\partial^{2}l}{\partial \theta \partial p}=\frac{\partial^{2}l}{\partial p\partial \theta }. \end{array}$$
